# Perceived Stress, Resilience and Emotional Intelligence in Romanian Healthcare Professionals

**DOI:** 10.3390/healthcare12232336

**Published:** 2024-11-22

**Authors:** Lavinia Duică, Elisabeta Antonescu, Maria Totan, Oana Raluca Antonescu, Gabriela Boța, Ionela Maniu, Mihail Cristian Pirlog, Sînziana Călina Silișteanu

**Affiliations:** 1 Faculty of Medicine, “Lucian Blaga” University of Sibiu, 550169 Sibiu, Romania; lavinia.duica@ulbsibiu.ro (L.D.); maria.totan@ulbsibiu.ro (M.T.); oanaraluca.antonescu@ulbsibiu.ro (O.R.A.); gabriela.bota@ulbsibiu.ro (G.B.); 2 “Dr. Gh. Preda” Clinical Psychiatric Hospital of Sibiu, 550082 Sibiu, Romania; 3 County Clinical Emergency Hospital of Sibiu, 550245 Sibiu, Romania; 4 Clinical Hospital for Children of Sibiu, 550164 Sibiu, Romania; 5 Faculty of Sciences, “Lucian Blaga” University of Sibiu, 550012 Sibiu, Romania; ionela.maniu@ulbsibiu.ro; 6 Department of Medical Sociology, Faculty of Medicine, University of Medicine and Pharmacy of Craiova, 200349 Craiova, Romania; mihai.pirlog@gmail.com; 7 Faculty of Medicine and Biological Sciences, “Ștefan cel Mare” University of Suceava, 720229 Suceava, Romania

**Keywords:** stress, resilience, emotional intelligence, healthcare professionals, education

## Abstract

Background: Occupational stress and burnout in the medical field are common factors that can have a negative impact on the quality of clinical care. In the Romanian healthcare environment, there exists important financial difficulties contributing additionally to stress in this study, we aimed to investigate if resilience and emotional intelligence would prove to be protective factors against stress. Methods: In our cross-sectional study, we investigated 189 medical professionals, using convenience sampling, from July 2022 to September 2022 in two university centers. We applied a self-reported questionnaire that included socio-demographic characteristics and three scales that measured perceived stress (the Perceived Stress Scale), resilience to stress (the Connor–Davidson Resilience Scale), and emotional intelligence (the short-form Trait Emotional Intelligence Questionnaire). Results: Age was positively associated with resilience levels, meaning that resilience increases with age. While specialist physicians had the highest emotional intelligence score, nurses and other healthcare workers had the highest resilience scores. Perceived stress level was negatively correlated with resilience to stress and with emotional intelligence levels. Resilience to stress was positively correlated with emotional intelligence. Conclusions: The major strength of this study is the finding that resilience to stress mediated the association between perceived stress and emotional intelligence. Because resilience is negatively associated with burnout, resilience to stress and emotional intelligence are potential targets for training aimed at improving the working environment and reducing current levels of burnout in the Romanian Health System and beyond.

## 1. Introduction

Perceptions of stress comprises medical, physical, psychological, and psychosocial aspects in the healthcare environment [1]. A number of stressors are associated with health professions, including a great deal of stress, increased pressures, and problems related to healthcare management [2]. Apart from these general stress factors, nursing is associated with complex work demands and excessive responsibilities coupled with minimal authority [3], while physicians deal with working conditions, long years of training, a lack of balance between work and their personal lives, and the legal implications of their decisions [4]. It is important to prevent stress in medical personnel since stress is known to be an important contributor to cardiovascular disorders [5].

The limited financial resources in the Romanian Health System have led to various challenges in multiple fields. Healthcare expenditure in 2021 in Romania, mostly centered on hospitals, is at the bottom of the European rankings: 6.3% of the GDP [6]. Regarding human resources, the number of medical personnel is relatively low in Romania compared to the healthcare workforce in Europe (3.2 doctors per 1000 population, while the EU average is 3.9 and 7.5 nurses per 1000 population compared with the EU average of 8.4) [7]. Although the education system in Romania trains large numbers of medical students and nursing graduates, in recent decades, many of them have emigrated to EU countries due to higher salaries and better working conditions [8].

Under these conditions, it is necessary to develop new methods to improve professional performance. Among these, the training of human resources to equip them with protective mechanisms against occupational stress can be a viable way to obtain better professional results.

A number of studies have shown that resilience and coping strategies had both direct and mediating effects of stress on medical personnel, thereby serving as protective factors [9,10], and the emotional intelligence of healthcare professionals determines the quality of the relationship between the healthcare provider and the patient [11].

The results of research programs have stated that resilience has a significant negative correlation with perceived stress in medical staff [12,13,14]. Resilience refers to the protective mechanism of an individual who has suffered traumatic events in the past, contributing to the development of coping abilities [15]. It represents a factor that protects the individual experiencing stress [16] and it prevents burnout and can improve patient outcomes [17]. Resilience mediates the relationship between the perceived stress and psychological distress symptoms as well as anxiety and depression [18]. Increased resilience may reduce stress via psychological mechanisms and physical exercises [19,20].

Studies conducted on medical students revealed a significant negative correlation between perceived stress and the level of emotional intelligence [21,22]. From Goleman’s perspective, emotional intelligence is the capacity to manage one’s own emotions, as well as those of others, in order to achieve better results at work [23]. Emotional intelligence reduces stress, and conversely, it disappears under extreme stress [24].

In Saddki’s study, the role of emotional intelligence as an independent predictor of perceived stress was demonstrated [25]. Further, as emotional intelligence enables an individual to properly evaluate the environment and organize thoughts according to the priority of problems, it increases resilience [26].

The following hypotheses are outlined: 1. Emotional intelligence and resilience to stress are negatively correlated with perceived stress on the one hand, and on the other hand, resilience to stress and emotional intelligence are positively correlated with one another. 2. There are significant differences between the demographics, type of profession (specialist physicians, resident physicians, and nurses), and resilience and emotional intelligence levels. 3. Resilience acts as a mediator between perceived stress and emotional intelligence.

The aim of this study is to evaluate the levels of perceived stress, resilience to stress and emotional intelligence, and the relationships between them among medical staff in Romania. Although previous studies have investigated this type of interrelation, this paper will assess the characteristics of these relationships in Romanian medical personnel in terms of demographics or working characteristics. We will also analyze whether a causal chain exists among the variables studied, the temporal precedents of exposure (perceived stress), mediators (resilience to stress), and outcomes (emotional intelligence).

## 2. Materials and Methods

### 2.1. Participants and Data Collection

Questionnaires were distributed personally in medical wards, and chief physicians were asked to allow their staff to complete them. A sample of 252 health professionals was recruited for this study, using convenience sampling. Amongst them, 189 submitted responses, 34 did not provide complete responses, and 29 did not submit responses, citing a lack of time. We analyzed this sample of 189 healthcare professionals in two university centers (Sibiu and Suceava) in a cross-transversal study. The data used were parts of information collected from a range of medical staff, including doctors, residents, nurses, and other health workers. The collection period was between July 2022 and September 2022.

We collected data from the individuals in the medical wards through face-to-face interviews. Each participant in this study was asked to complete a questionnaire. The questionnaire included two sections: (1) one section with open-ended questions asking about the socio-demographic characteristics (gender, age, marital occupation, marital status, and place of residence) and (2) the second section containing three self-administered scales measuring the perceived stress (the Perceived Stress Scale—PSS), resilience to stress (the Connor–Davidson Resilience Scale—CD-RISC 25), and emotional intelligence (the short-form Trait Emotional Intelligence Questionnaire—TEIQue-SF). Informed written consent was obtained from each participant in this study.

The Perceived Stress Scale (PSS) [27] is a 10-item measure of self-appraised stress. The PSS is related to a number of psychological responses, including anxiety and depressive symptoms [28]. A total PSS score ranging from 1 to 13 would be considered low stress, a score ranging from 13 to 26 would be considered moderate stress, and a score over 27 would be considered high perceived stress [27]. The PSS-10 has a Cronbach’s α value of 0.78, demonstrating good reliability [27].

The Connor–Davidson Resilience Scale (CD-RISC) is a 25-item scale that measures resilience to stress (RS) [15]. Resilience intervenes in many types of situations, as a response to mild or intense psychotraumatic factors [29]. Personal factors [30], biological factors [31], and environmental factors (e.g., social support) can contribute to RS [32]. The CD-RISC is a 25-item scale with a Cronbach’s alpha value of 0.89 in the general population, indicating good internal reliability [15]. The CD-RISC is one of the most regularly used scales due to the high quality of its psychometric properties [33]. Beside these properties, in this study we chose this scale among several other scales because CD-RISC is based on Connor and Davidson’s operational definition of resilience. Additionally, previous studies indicate that CD-RISC significantly negatively correlated with the Perceived Stress Scale [15].

The short-form Trait Emotional Intelligence Questionnaire (TEIQue-SF) measures emotional intelligence (EI). This is a questionnaire containing 30 items and is the short form of the full questionnaire, which consists of 153 items [34]. Its construction (including 15 facets, associated with 4 factors) produces a trait global score and is based on EI theory [35]. Petrides defined these four factors as well-being (overall well-being), self-control (regulating stress and impulses), emotionality (the capacity to perceive and express emotions), and sociability (the capacity to socialize). The overall Cronbach α of the questionnaire is high (α = 0.925) [36]. For the present study, we consider the trait emotional intelligence (trait EI) model because it is the most widely researched [37] and has been studied in numerous studies in multiple contexts and the association with other scales, which have indicated that trait EI is a positive predictor of well-being and is negatively associated with job stress and burnout [38].

In this study, the Cronbach’s α coefficient for the PSS, CD-RISC-25, and TEIQue-SF were 0.833, 0.909, and 0.844.

All the subjects in this research were informed about the purpose of this study. All study procedures were approved by the Human Research Ethics Committee of “Stefan cel Mare” University in Suceava under protocol number 49/21/2022.

### 2.2. Data Analysis

Data analysis was performed using Statistical Package for Social Sciences v.20. Numbers and percentages, means (Ms) and standard deviations (SDs), and medians and interquartile ranges (IQRs) were used to describe the variables. A comparison of the PSS, RS, and EI scores was conducted using Student’s *t*-test, one-way ANOVA, or the Kruskal–Wallis test, and the post hoc Bonferroni and Dunn test. The normal distributions of the continuous variables were checked using skewness, kurtosis, and Shapiro–Wilk test. Correlations among the variables were assessed by calculating Pearson’s correlation coefficients. The reliability of the questionnaires was evaluated using Cronbach’s alpha. To detect CMV (common-method bias), Harman’s one-factor test was used (percentage of variance for a factor less than 50%, indicating no serious common-method bias). A *p* value less than 0.05 was considered statistically significant.

Mediation analysis was used to explore the relationship between EI, RS, and the PSS. We considered a model of mediation with EI as a predictor, RS as a mediating variable, and the PSS as dependent variable; perceived stress is caused by emotional intelligence with some of this impact being mediated by resilience. The path coefficients represent the beta (β) coefficients of the regression models between the included variables.

The coefficient *a* represents the direct relation between EI and RS, the coefficient *b* represents the relation between EI and the PSS, the coefficient c represents the direct effect path between EI and the PSS, and c’ represents the mediated effect, i.e., the effect of EI on the PSS controlling for RS. The mediated effect is estimated by the mediated effect is estimated by multiplying coefficients a and b [39]. For the estimation of the mediation model, a path analysis–structural equation modelling (SEM) procedure was performed through the use of the Iavaan R package [40]. The significance of the mediating effect was assessed using the non-parametric bootstrap procedure (5000 iterations).

## 3. Results

The demographics (age, gender, and marital status) and working characteristics (department and working occupation) of the subjects are reported in Table 1. In our sample, a majority of those who responded were female (78.3%), and the mean age was 41.19 years (SD = 10.56); 31.7% of the healthcare professionals were in the age group 25–34 years old, most of them were married (65.6%), and in terms of medical profession, a majority of them were nurses (51.3%).

Table 1 shows that no statistically significant difference was found between the demographic characteristics of the subjects and the PSS and EI scores. There was a positive correlation between RS levels and age (r = 0.224, *p* = 0.002), with older age groups higher in resilience (*p* = 0.011).

In terms of work characteristics, profession was significantly positively related to the level of RS and positively correlated with the resilience score (*p* = 0.003) and level of EI (*p* = 0.000).

**Figure 1:**
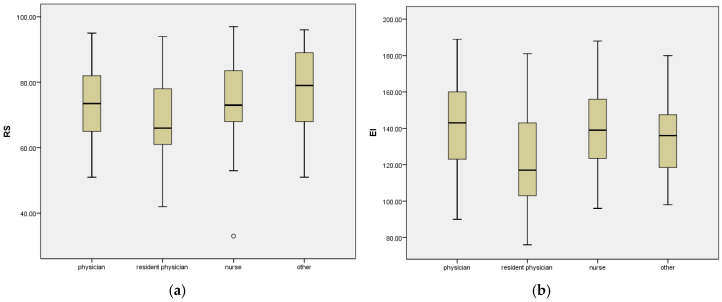
(**a**) RS and (**b**) EI scores by profession. Significant differences (post hoc test) were encountered (**a**) between nurses and resident physicians (*p* = 0.009), between other healthcare workers and resident physicians (*p* = 0.003), (**b**) between physicians and resident physicians (*p* = 0.001), and between nurses and resident physicians (*p* = 0.000).

The resident physicians had the lowest RS scores and the lowest EI scores, while the specialist physicians had the highest EI scores. The nurses and other healthcare workers had higher RS scores compared to the resident physicians (*p* = 0.009; *p* = 0.003). The specialist physicians and nurses had higher EI scores compared to the resident physicians (*p* = 0.001; *p* = 0.000) (Figure 1a,b).

The mean score for the PSS was 15.51 (SD = 5.89). A majority of the sampled health professionals perceived moderate levels of stress (57.7%), and only 3.1% of them perceived high levels of stress (Table 2). For the RS, the mean score for the CD-RISC-25 was 74.03 (SD = 12.25), the median was 73, and the IQR was between 66 and 84. The item mean score varied between 2.16 (SD = 1.02: item 18) and 3.41 (SD = 0.66: item 1). The low resilience scores (<66) corresponded to the first quartile (Q1) (25.5%), the moderate resilience scores (66–72 and 73–84) were in correspondence with the second and third quartiles (Q2 and Q3) (23.4% and 26.1%), respectively, and the highest resilience scores (>84) corresponded with the fourth quartile (Q4) (Table 2). In the case of EI, the mean score was 136.39 (SD = 23.58), the median was 136, and the IQR was between 120 and 155. A total of 40% of the participants indicated average EI scores (123–148), while 30% of them had high EI scores (Table 2).

Table 3 shows the correlations among the PSS, RS, and EI scores, showing that the PSS was negatively correlated with both RS (r = −0.376, *p* < 0.001) and EI (r = −0.465, *p* < 0.001). RS was positively correlated with EI (r = 0.490, *p* < 0.001). The greater the perceived stress experienced by the individual, the less they were able to manage their emotions.

The PSS correlated negatively with the subscales of EI; the most negative correlation was between the PSS and well-being subscale (−0.479) (facets: Happiness, Optimism, and Self-esteem) and self-control subscale (−0.482) (facets: Emotional regulation, Stress management, and Impulsivity). RS correlated positively with the subscales of EI; the highest positive correlation was between RS and the self-control subscale, too (0.520).

**Figure 2:**
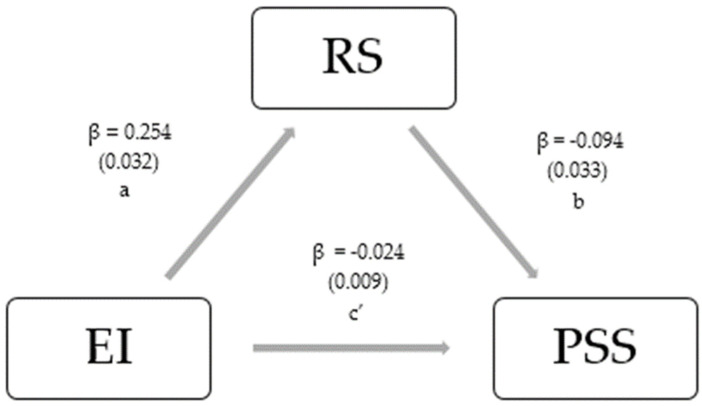
Mediation of RS relating to the association between EI levels and PSS levels. The results are presented as beta (β) standardized coefficients (standard error).

The mediation model of resilience relating to the association between EI and the PSS is depicted in Table 4 and Figure 2. The model showed that RS mediated the association between EI and the PSS (*p* = 0.011). There was a significant positive association (path a) between EI and RS (β = 0.328, *p* < 0.001). The path coefficient between RS and the PSS (path b) was statistically significant and negative (β = −0.093, *p* = 0.047). The indirect effect (path c’) was negative and significant (β = −0.024, 95% CI (−0.044; −0.007), *p* = 0.011).

## 4. Discussion

In the current study among Romanian healthcare professionals, the demographic parameters (age, gender, and marital status) were not significantly related to the level of PSS or EI levels; instead, one demographic parameter—age—was significantly statistically positively associated with RS levels. The mean score for the PSS, RS, and EI indicated moderate levels. Comparing the personnel categories, the results indicated that the nurses and other healthcare workers had a higher resilience score compared with the medical doctors, and the physicians had the highest emotional intelligence scores. The resident physicians indicated the lowest RS and EI scores. The PSS and RS indicated moderate levels, and 40% of the participants had moderate EI levels. The PSS was negatively correlated with both RS and with EI, and more importantly, RS correlated positively with EI, under the conditions in which RS mediated the association between EI and the PSS.

Some previous studies have revealed some associations between PSS or EI scores and demographic parameters. In a literature review, associations between PSS or EI scores and demographic parameters showed different results. Yada et al. found that, for Japanese nurses, men had significantly lower stress levels than women [41], while other studies conducted in Spain and Jordan indicated higher PSS levels in females in the younger age groups [42] and higher stress scores among single nurses compared to married nurses [43]. In Turkey, for nurses in the 20–25 year age group, the mean PSS scores were statistically significantly higher those of other groups [44]. A study carried out by Chang found no association between Malaysian nurses’ EI levels and their gender or marital status [45]. In a study performed by Aljboa et al., in Saudi Arabia, age was found to be significantly related to EI, specifically for older nurses [46]. It should be “Higher scores for EI have been significantly associated with healthcare experience, as in a previous study, whereby Turkish family physicians over the age of 29 years had the highest mean values for EI [47]”.

In this study, RS scores increased with age, and the most resilient healthcare professionals were over 55 years old. A study carried out in Turkey on healthcare personnel revealed a significant positive association between RS and age [48]. A study carried out by Eley et al. in Australia on physicians found that age was significantly statistically associated with RS levels [49]. Other studies have shown different results; for example, in a study conducted on Australian nurses, there was no significant association between age and RS scores [50], and in Hong Kong, junior nurses demonstrated resilience in that facing obstacles led to psychological growth [51].

In a study by Hsieh et al. (2024) [52] that investigated adults with a mean age of 41.79 ± 16.99 years, older adults demonstrated greater subjective well-being, an outcome mediated by resilience to stress. In older adults (age ≥ 55 years), as compared to young adults, amygdala function was associated with pronounced positivity over negative information in attention and memory [53] or decreased attentional bias to negative stimuli [54]. The positivity effect could be caused by increased amygdala habituation [55] or explained by the socioemotional selectivity theory, which posits that the prioritization of emotional goals and more positive emotional experiences come with increased age [56]. Older adults have been shown to be more resilient in terms of emotional regulation and problem-solving [57]. Furthermore, acceptance is a self-regulation strategy related to resilience that increases with age [58]. As it is considered an adaptive strategy for increasing long-term well-being, acceptance could prevent professional burnout in nurses [59]. The accumulation of life experiences leads to a shift in personality maturation and acquisition of emotional stability [60]. Nurses with emotional stability or less reactivity tend to have more competence in problem-solving [61].

In terms of working characteristics (profession and medical specialty), this study showed that resident doctors indicated the lowest RS and EI scores in comparison with all other categories of personnel (specialist doctors, nurses, and other healthcare professionals). Resident doctors all over the world have a tremendous workload and a great amount of responsibilities. In a study by McKinley et al., resident doctors in the United States of America scored low on social awareness and adaptability, two characteristics that would be worth considering in a medical profession. Low adaptability could be an expressed by a low tolerance toward change and uncertainty, and this may be possible explanation for the results of our study [62]. In Romania, the cause of this relationship could be explained by the instability of their living conditions. It is well known that many resident and young doctors migrate extensively to European countries in search of better training and higher wages [63].

Moreover, in the present study, the results show that nurses and other healthcare workers had a higher resilience score compared with medical doctors. On the contrary, other findings have suggested that, in the United States, medical doctors have higher resilience scores than nurses [64]. In an important survey study in the United States, RS scores were the highest for doctors in some surgery departments such as neurosurgery and in emergency wards, and they were the lowest for doctors in neurology and pediatrics [65]. The increased RS scores of nurses and other healthcare workers compared with those of physicians could be due to personal training or job characteristics, poor working conditions, and very high responsibility in relation to patients. Additionally, the results of this study noted that physicians had the highest emotional intelligence scores, as opposed to a study from the United States that found no significant differences between the surgeons and trainee surgeons and that nurses had higher EI scores than surgeons [66].

The association between PSS and EI scores and working characteristics has been the subject of previous studies. In a study performed in Pakistan, the association between physicians’ perceived stress levels and their specialty was not significant [67]. In Ireland, PSS scores were significantly higher for nurses working in emergency departments (emergency rooms and intensive care) as well as for nurses in medical and pediatric departments than they were for nurses in other wards [68]. Cokun et al. found lower EI scores for men than for women among family physicians [47] in Turkey. On the contrary, Aghajani et al. reported higher EI scores for men compared to women among Iranian medical students [69].

In the present study, the mean PSS score was 15.51 ± 5.89, and we found that a majority of the medical personnel (57.7%) had moderate levels of stress, and 3.1% of the participants had severe stress levels. A series of past studies has demonstrated higher levels of stress than that found by us in the present study. Most of the doctors in a hospital in Bengal had moderate levels of stress (80%), while 9.5% had severe stress levels. In another study in Bengal, the mean PSS score attained was of a moderate level at 20.49 ± 5.6 [70]. In a study in Poland, 52.6% of the nurses sampled had average PSS scores, and 24.1% had high PSS scores [71]. In a study conducted in Jordan, the mean PSS score for residents was 21.6, and 73% of the medical residents had moderate levels of stress—a fairly high proportion—while 18% had high levels of stress [72]. A study conducted in Saudi Arabia reported that the mean PSS score for resident trainees was 20 ± 5.51, and of these, approximately 83% indicated moderate levels of stress, while 10% of the medical residents indicated high levels of stress [73].

The mean for CD-RISC-30 score in the present study indicated moderate levels in 60.5 of cases (the RS score was 73.61 ± 11.89 for specialist physicians, 67.42 ± 11.81 for resident physicians, and 75.15 ± 11.76 for the nurses). Slightly lower mean scores have been indicated in some other studies. In a sample of New York doctors, 63.9% of the participants had high RS scores [74]. Moderate levels of resilience were found for Spanish nurses [18] and Iranian nurses [75]. A survey in French hospitals that evaluated physicians working in the emergency department, anesthesiology, and infectious disease departments, as well as the intensive care unit, found a median RISC-25 score of 69, a moderate score [76]. Quite similar percentages have been documented in a study conducted on newly hired nurses in Jordan during the COVID-19 pandemic; the mean RS score was 74.98 ± 14.01, and the moderate-to-high-resilience group (Q3 and Q4) represented 52% of the participants [77].

The results of this study showed a mean EI score of 136.39 ± 23.58 (the EI score was 141.96 ± 23.05 for specialist physicians, and it was 140.89 ± 21.40 for nurses), 40% of the personnel had moderate EI levels, and 30% of the sampled medical personnel had high EI scores. These scores where higher than in other studies. Mean EI scores were very low in a study conducted by Amini et al. in Teheran, where healthcare staff had a mean EI of 76.49 ± 2.83 and nurses had a mean score of 73.81 [78]. In a study conducted among doctors in South India, the total EI score obtained was 116.08 ± 14.76; 72.9% of the doctors had average EI scores, and 12.9% had good EI scores [79]. In that study, the mean EI score was 117.07 ± 27.87, which was lower than the mean EI score for the entire medical personnel sample, but was higher than that found in a study performed at Harvard Medical School where the mean EI score for residents was 101.0 ± 8.1 across three separate residency programs (surgery, pediatrics, and pathology) [80]. Furthermore, in a study in Lithuania, the average EI questionnaire score was 147.8 ± 26.86 for medical residents [81]; this is higher than the EI score found in the current study among the medical residents (120.39 ± 26.44).

The PSS negatively correlated with both RS and EI in the current study. The presented results are consistent with previously published results. During the COVID-19 pandemic, a series of studies showed that the PSS was negatively associated with RS among healthcare workers in China [82], among critical care nurses in Saudi Arabia [83], as well as among Chinese frontline healthcare professionals [84]. For resident doctors in India, the PSS showed a negative correlation with EI [85]. The PSS had a negative correlation with EI among resident doctors in West Bengal [86]. In a sample of Greek healthcare professionals in mental health institutions, a significant negative relationship between EI and job stress was found [87]. A negative relationship between EI and the PSS was found among emergency medicine physicians in Turkey [88]. An inverse correlation between EI and self-reported stress was found for Korean nurses, and it was also found that online mind–body training programs and other physical exercises could decrease stress [89,90].

An important result of this study is that RS correlated positively with EI, and the most significant positive association is found between RS and the well-being subscale. These results are due to the fact that the ability to read the emotions of others and the capacity to regulate one own’s emotions and those of others are in accordance with own’s resilience to stress. Previous studies have published results that align with those of this study. During the COVID-19 pandemic, RS scores among staff nurses were found to have a moderately positive relationship with their EI score [46]. In Spain, during the COVID-19 pandemic, for healthcare workers, and especially for nurses, a past study’s findings suggested a significant positive relationship between RS and EI [91].

In the present study, the results show that RS mediated the association between EI and the PSS. This result is consistent with the findings from a study performed on psychology students from the United States and Northern Spain, where EI and the PSS were linked through resilience, and where emotional intelligence was a negative predictor of perceived stress through resilience [92]. This is an important result of this study because it reflects the construct validation of the concept of resilience. According to the new definitions of resilience following intense debates on the validity of the concept of resilience in its first forms, it emerged that it can be considered a process by which an individual manages significant adversities well [93]. So, resilience would mediate the production of positive outcomes in the presence of adversity. By extrapolation, in this study, adversity and positive outcomes can be equated with perceived stress and emotional intelligence, respectively, and the results of this study point out that resilience mediates between adversity (perceived stress) and positive outcomes (emotional intelligence) [94].

A limitation of the present study is its small sample size due to resource constraints. Due to the limited resources, there were many incomplete responses. A multicentric study with a large sample size could provide other types of information, such as a comparison of the PSS, RS, and EI scores for different professional categories across a wider variety of medical and surgical wards, as well as emergency care units. The convenience sampling method used in this study could be a source of biases in terms of external validity, because the sample may not be representative of the population of medical personnel. Another limitation of this study is the fact that, in Romania, there are no other studies that measure the mentioned variables; at the least, there are no studies that use the same instruments. This is why, in Section 4, we could not refer to previous studies on Romanian medical staff. Moreover, in the future, we aim to evaluate the same psychological characteristics in a longitudinal study before and after educational interventions using emotional intelligence training.

## 5. Conclusions

The present study demonstrated a negative association between emotional intelligence and the resilience of medical staff and the stress perceived by them. In light of the results, the idea supported is that emotional intelligence and resilience are considered a protective mechanism against stress.

In other words, being resilient and emotionally intelligent could provide nurses and doctors the ability to avoid burnout or occupational stress and improve their job satisfaction.

Since resilience acted as a mediator in the relationship between emotional intelligence and perceived stress for healthcare professionals, in general, it may be appropriate to concentrate efforts on improving resilience within the workplace.

Higher resilience could prevent burnout and contribute to a good professional quality of life in healthcare staff.

In the Romanian Health System, the limited financial and human resources could be countered through introducing procedures for training resilience and emotional intelligence in nurses, doctors, and other healthcare workers, to enhance professional and personal outcomes and prevent emotional strain and burnout.

## Figures and Tables

**Table 1:** Comparisons of the PSS, RS, and EI scores, the demographics, and the working characteristics of the sampled population.

		n = 189 (%)	PSS M ± SD	*p*	RS M ± SD	*p*	EI M ± SD	*p*
Gender	Male	41 (21.7)	15.00 ± 6.25	0.529	71.00 ± 13.17	0.073	133.73 ± 25.50	0.414
	Female	148 (78.3)	15.66 ± 5.80		74.88 ± 11.90		137.14 ± 23.05	
Age								
	18–24	5 (2.6)	16.60 ± 3.13	0.992	68.60 ± 12.34	**0.011**	127.40 ± 10.92	0.153
	25–34	60 (31.7)	15.32 ± 6.57		69.97 ± 12.09		133.98 ± 26.47	
	35–44	45 (23.8)	15.52 ± 5.89		74.91 ± 12.58		131.86 ± 22.03	
	45–54	58 (30.7)	15.65 ± 5.48		77.53 ± 11.29		140.63 ± 23.53	
	≥55	21 (11.1)	15.43 ± 5.89		75.57 ± 11.92		143.43 ± 17.34	
Marital status	Married	124 (65.6)	15.24 ± 5.67	0.675	74.77 ± 11.92	0.469	137.35 ± 22.67	0.391
	Unmarried	57 (30.2)	16.02 ± 6.58		72.86 ± 12.45		135.70 ± 25.93	
	Widowed	8 (4.2)	16.29 ± 3.99		70.57 ± 16.91		125.00 ± 19.18	
Profession	Specialist physician	28 (14.8)	14.50 ± 6.33	0.073	73.61 ± 11.89	**0.003**	141.96 ± 23.05	**0.000**
	Resident physician	33 (17.5)	16.97 ± 6.29		67.42 ± 11.81		120.39 ± 26.44	
	Nurse	97 (51.3)	14.76 ± 5.11		75.15 ± 11.76		140.89 ± 21.40	
	Other healthcare worker	31 (16.4)	17.19 ± 6.87		78.00 ± 12.40		134.61 ± 20.29	

n = number of participants in this study; M = mean; SD = standard deviation; *p =* probability value; PSS = Perceived Stress Scale; RS = resilience to stress; EI = emotional intelligence; bold *p*-values mean *p*-values less than 0.05 (*p* ≤ 0.05).

**Table 2:** Means, standard deviations, and levels of PSS, RS, and EI scores.

Scales	Mean ± SD	PSS Score	%	RS Score	%	EI Score	%
PSS	15.51 ± 5.89	Low (≤13)	39.2	Low (≤66) (Q1)	25.5	Low (≤122) (1–30%)	30
RS	74.03 ± 12.25	Moderate (14–26)	57.7	Moderate (66–83) (Q2, Q3)	60.5	Average (123–148) (31–70%)	40
EI	136.39 ± 23.58	High (≥27)	3.1	High (≥84) (Q4)	25	High (≥149) (71–100%)	30

**Table 3:** Pearson’s correlation coefficients between the PSS, RS, EI, and subscales of EI.

	RS	EI	EI Subscales			
			Emotionality	Sociability	Well-Being	Self-Control
**PSS**	−0.376 **	−0.465 **	−0.313 **	−0.348 **	−0.479 **	−0.482 **
**RS**		0.490 **	0.309 **	0.425 **	0.446 **	0.520 **

** *p* ≤ 0.01.

**Table 4:** Model pathways where EI is the independent variable, RS is the mediator, and the PSS is the dependent variable.

Model Pathways	Standardized β	Std. Error	z	*p*	95% CI	
					Lower	Upper
EI → RS	0.254	0.032	7.887	0.000	0.190	0.318
RS → PSS	−0.094	0.033	−2.833	0.005	−0.162	−0.029
EI → PSS	−0.092	0.018	−5.163	0.000	−0.128	−0.057
EI → RS → PSS	−0.024	0.009	−2.528	0.011	−0.044	−0.007

Std. Error = standard error; β = coefficient β; z = z-score; *p* = probability value; CI = confidence interval.

## Data Availability

The data presented in this study are available on request from the corresponding author.
